# Preprints: An underutilized mechanism to accelerate outbreak science

**DOI:** 10.1371/journal.pmed.1002549

**Published:** 2018-04-03

**Authors:** Michael A. Johansson, Nicholas G. Reich, Lauren Ancel Meyers, Marc Lipsitch

**Affiliations:** 1 Outbreak Science, San Juan, Puerto Rico, United States of America; 2 Department of Biostatistics and Epidemiology, University of Massachusetts, Amherst, Massachusetts, United States of America; 3 Department of Integrative Biology, University of Texas at Austin, Austin, Texas, United States of America; 4 Center for Communicable Disease Dynamics, Department of Epidemiology, Harvard T.H. Chan School of Public Health, Boston, Massachusetts, United States of America

## Abstract

In an Essay, Michael Johansson and colleagues advocate the posting of research studies addressing infectious disease outbreaks as preprints.

**Summary points**

- Preprints—manuscripts posted openly online prior to peer review—offer an opportunity to accelerate the dissemination of scientific findings to support responses to infectious disease outbreaks.
- Preprints posted during the Ebola and Zika outbreaks included novel analyses and new data, and most of those that were matched to peer-reviewed publications were available more than 100 days before publication.
- Despite the advantages of preprints and the endorsement of journals and funders in the context of outbreaks, less than 5% of Ebola and Zika journal articles were posted as preprints prior to publication in journals.
- With broader adoption by scientists, journals, and funding agencies, preprints can complement peer-reviewed publication and ensure the early, open, and transparent dissemination of science relevant to the prevention and control of disease outbreaks.

Emerging public health threats, such as infectious disease outbreaks, require swift and evidence-based responses informed by science. However, the communication of scientific research is notoriously slow [1]. When rapid dissemination of new information has the chance to prevent or control epidemics affecting hundreds or thousands of people, we must fast-track this process. Preprints (manuscripts posted publicly prior to peer review) have been endorsed as a solution to this challenge, yet adoption remains very low and needs to be improved.

On February 10, 2016, more than 30 of the world’s largest and most prestigious public health journals and funding agencies issued a landmark statement on the importance of preprints and data sharing in public health emergencies such as the Ebola and Zika epidemics [2]. Journal signatories pledged to (1) make related scientific content freely accessible, and (2) allow data and preprint manuscripts to be shared prior to publication. Here, we focus on preprints, which may include both new data and new analyses and offer an opportunity to speed and democratize further scientific analyses and the availability of evidence to inform outbreak responses.

The statement coincided with a proliferation of Zika research in response to the epidemic in the Americas (Fig 1A). Between November 2015 and August 2017, we identified a total of 174 preprint manuscripts with Zika in the title or abstract in 5 recognized public preprint repositories, including 4 general repositories: bioRxiv (124, http://www.biorxiv.org/), arXiv (31, arxiv.org), F1000Research (12, with 1 other that was originally posted in bioRxiv, f1000research.com), PeerJ Preprints (5, peerj.com/preprints/), and the World Health Organization Zika Open repository (2, www.who.int/bulletin/online_first/zika_open/), which was established explicitly for the Zika epidemic (S1 Dataset). These likely represent the majority of Zika preprints, though others may have been posted in ad hoc, lesser-known, or laboratory- or university-specific webpages or repositories. Over a similar time period in the Ebola outbreak (May 2014 to January 2016), there were 75 Ebola preprints (Fig 1B). There were many more publications in peer-reviewed journals over these time periods: 1,641 and 2,187 publications indexed in PubMed (http://www.ncbi.nlm.nih.gov/pubmed, S2 Dataset) with Ebola or Zika, respectively, in the title or abstract and of type “Journal Article” (257 and 417 additional publications were classified as letters, reviews, etc., respectively). This increase in publications (33%) cannot explain the increase in preprints alone (132%). Over the same time period, there was a general increase in preprints for health sciences [3]; for example, bioRxiv submissions with the word “outbreak” rose approximately 10-fold between 2014 (24) and 2016 (232). Thus, the increase in preprints related to the Zika epidemic likely reflects this general trend, some increase in research, and possibly the influence of the statement on data sharing.

**Fig 1:**
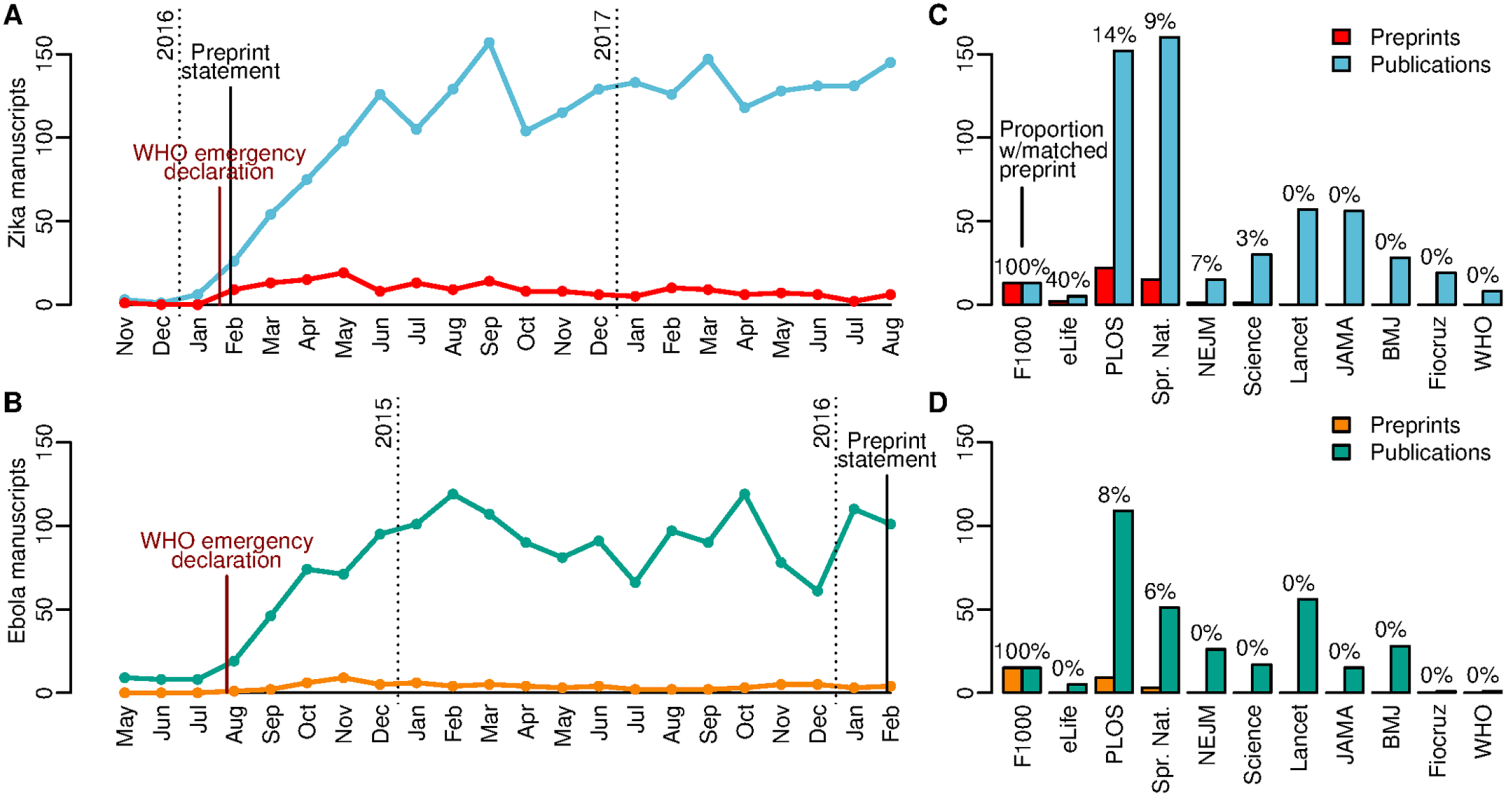
Zika and Ebola outbreak preprints and publications. Panels A (Zika) and B (Ebola) show the total number of newly posted preprints (arXiv, bioRxiv, F1000Research, PeerJ Preprints, or World Health Organization Zika Open) and journal articles (PubMed indexed) by month. Dates were the initial submission date for preprints and the journal publication date for PubMed (or the PubMed entry creation date if a specific journal publication date was not supplied). Panels C (Zika) and D (Ebola) show the number of preprints and publications by publisher for each of the publishers who were signatories to the data sharing statement ordered by the proportion of publications with a matched Zika preprint. Publisher abbreviations: BMJ, The British Medical Journal; F1000, Faculty of 1000; Fiocruz, Fundação Oswaldo Cruz; JAMA, The Journal of the American Medical Association Network; NEJM, The New England Journal of Medicine; PLOS, Public Library of Science; Science, Science Journals; Spr. Nat., Springer Nature; WHO, Bulletin of the World Health Organization.

To assess changes in preprint posting during the 2 epidemics, we examined the subset of all preprints that could be matched with an eventual publication. For Ebola, we matched 45 (60%) of the preprints to PubMed-indexed journal articles, and for Zika, 84 (48%). Four additional Zika preprints were matched to “Review” (1) or “Comparative Study” (3) publications in PubMed. Unmatched preprints may be preprints that were never submitted for peer-reviewed publication (e.g., opinions or preliminary work that was abandoned), were never accepted for publication, or were still in peer review at the end of the selected time periods.

Preprints were increasingly used to disseminate new data during the Zika outbreak. Among the subset of preprints matched to journal articles described above, the proportion of preprints including original data increased substantially from 7% for Ebola to 46% for Zika (difference: 40%, 95% confidence interval [CI] 25% to 54%, 2-sample test of proportions). While a minority of preprints contained new data, the majority of preprints in both outbreaks included novel analyses, 84% for Ebola and 94% for Zika (estimated difference: 10%, 95% CI −4% to 23%). The remainder were comprised of opinions, proposals for new lines of study, or research indirectly related to Ebola or Zika. The increase in data sharing between epidemics appears to represent a shift away from waiting for peer review and towards rapidly reported, open science. This shift should enable other researchers to build upon those findings and recognize the high value of data sharing (academic and otherwise), a common challenge in the midst of outbreaks.

Preprint posting also led to earlier access to those data and analyses. Excluding manuscripts in F1000Research, which uses the preprint posting date as the publication date, the median delay between preprint posting and publication was approximately 150 days for both outbreaks, and less than 25% of the manuscripts were published within 100 days of posting. Notably, this delay, which may include submission to multiple journals, is quite similar to normal publication timelines [1]. It is unclear to what extent journals are able to accelerate publication in outbreaks, but it is clear that every time there is an editorial or peer review decision, rejection, or revision there are delays, and that preprint posting precludes delays in broad access to the information.

The successes of preprints in the Ebola and Zika epidemics belie a more complex story about preprint use during these outbreaks. While the number of preprints increased, data sharing was more common, and scientific findings were available earlier, adoption remained extremely low. Publications with preprints represented a small minority of all PubMed-indexed journal articles, at approximately 3.4%. The proportion was slightly higher for Zika compared to Ebola (3.8% versus 2.7%), likely indicating a small increase between outbreaks (estimated difference: 1.1%, 95% CI −0.01% to 2.2%). Among signatory publishers, who represented approximately 25% and 29% of the publications for Ebola and Zika, respectively, this proportion was approximately 6.1% (95% CI 4.3% to 7.9%) higher than for nonsignatory publishers (7.8% versus 1.7%) (Fig 1C and 1D). This suggests that these publishers may have policies that are supportive of preprints irrespective of the statement [2].

Although preprint adoption in both outbreaks was very low, 4 important advances were clear. First, the relatively higher preprint usage for signatory publishers both before and after the statement indicates that policy supportive of preprints can encourage their use. Second, the number of preprints posted increased between the 2 outbreaks, likely reflecting changing attitudes towards preprints in the life sciences and changing policies, including the data sharing statement [4–6]. Third, preprints generally contained new analyses and increasingly shared novel data. Fourth, with preprints available months before peer-reviewed publications, preprint posting can accelerate the sharing of research.

Preprints also bring new challenges to outbreak responses [3]. By definition, preprints are not peer reviewed prior to posting. While preprint posting is common practice in fields such as physics and statistics, it is a new concept to many scientists in public health and even more so to public health officials, the press, and the public, all of whom may be seeking the latest information during epidemics. Until preprints are broadly recognized as pre-peer review manuscripts, they may be misinterpreted as peer-reviewed research. On the other hand, peer review faces its own challenges of subjectivity, bias, transparency, and speed [1,7,8]. Peer review is an integral component of scientific communication, but it does not intrinsically guarantee the quality of science. Moreover, peer review is particularly challenging during major outbreaks when the most qualified reviewers are also immersed in urgent research. Preprint posting may help mediate this process, providing an opportunity for broad and immediate community input that is not subject to the limits of traditional peer review [4–6]. Assuring ethical review, participant confidentiality, recognition of preprints as pre-peer review manuscripts, and finding mechanisms to enable transparent, open feedback will be essential to limiting possible negative impacts and maximizing the benefits of preprints for outbreak responses. Immediate, open access to research prior to peer review raises the possibility of misinterpretation and the misuse of science in critical decision making when lives are at stake, but it also permits early and open criticism, discussion, and consideration of findings that may save lives.

Further adoption of preprints also requires changes in how funders, scientists, and publishers value scientific contributions. All stakeholders agree that the best science should be brought to bear against outbreaks, yet they are also keenly aware of the importance of peer review and the scientific accolades that come with publishing novel, impactful research in prestigious journals. In the context of outbreaks, the goal of impacting the epidemic by immediately sharing important scientific findings conflicts with career goals tied to the slower, traditional, peer review-centered publication process. The low adoption of preprints during the Ebola and Zika epidemics is a symptom of uncertainty about preprints and this conflict of incentives. The signatory funders and publishers clearly endorsed preprints, but the majority of scientists did not realize that they could or should post preprints or thought that posting a preprint would jeopardize publication opportunities.

We advocate 4 steps to improve the adoption of preprints and speed the dissemination of science in the context of outbreaks. First, scientists should promote preprints by posting them and choosing to submit manuscripts to journals that accept preprints; many journals have policies that explicitly allow preprint posting (tools for identifying these journals are provided by Jisc: http://www.sherpa.ac.uk/romeo/search.php and Wikipedia: https://en.wikipedia.org/wiki/List_of_academic_journals_by_preprint_policy). Second, all publishers should endorse preprint posting for research related to outbreaks. This would send a clear signal to all scientists that preprints are integral to scientific communication and help nonscientists identify preprints as a distinct form of communication compared to peer-reviewed publications [1,4–6]. Publishers should actively encourage or require preprint posting at the time of submission, driving adoption directly as Faculty of 1000 has for F1000Research. Third, preprint repositories and the scientific community should ensure that preprints contain appropriate content (e.g., maintaining ethics and privacy) and are readily identifiable as preprints, and that mechanisms exist to facilitate community input prior to or concurrent with peer review. These considerations can help reduce the risks of preprints and maximize their benefits. Fourth, universities and funders should recognize preprints together with peer-reviewed publications and citations as an important part of an investigator’s track record, especially for any scientist involved in outbreak responses. The scientific community should not ask why preprints are posted during outbreaks, we should ask why they are not posted and make early posting the standard rather than the exception.

Preprints offer numerous challenges and opportunities for science in general but represent a particularly important opportunity to accelerate the dissemination of science in the midst of infectious disease outbreaks, when early actions are critical and evidence is scarce [9,10]. Despite this need and the 2016 statement on preprints and data sharing, less than 5% of Ebola and Zika journal articles were posted as preprints prior to publication in journals. This low adoption reflects an intrinsic and established, yet unnecessary, prioritization of the traditional publication process over the dissemination of science. Further progress is essential to ensure that science can be rapidly and broadly disseminated in the context of outbreaks. It is incumbent upon scientists, publishers, and funders alike to recognize the value of preprints and embrace them as a critical component of outbreak science.

## Supporting information

S1 Dataset Data on preprints included in the analysis. “preprint_date” is the initial preprint submission date from the respective repository. “pmid” is the PubMed identification number the preprint was matched to (if matched). “journal” and “pub_type” are from the PubMed entry. “publisher” indicates the publisher if the publisher signed the data sharing statement. “new_data” and “new_analysis” indicate whether the authors judged the manuscript to contain new data or analysis, respectively (1 = True).
(CSV)

S2 Dataset Data on PubMed journal articles included in the analysis. “pub_date” is the journal publication date as indicated by PubMed or the entry creation date if a specific journal publication date was not supplied. “pmid” and “journal” are the PubMed identification number and journal indicated by PubMed for the article. “publisher” indicates the publisher if the publisher signed the data sharing statement.
(CSV)

## References

[1] Powell K. Does it take too long to publish research? Nature. 11 February 2016. https://www.nature.com/news/does-it-take-too-long-to-publish-research-1.19320. Accessed 15 January 2018. 10.1038/530148a 26863966

[2] Wellcome Trust. Sharing data during Zika and other global health emergencies; 10 February 2016. https://blog.wellcome.ac.uk/2016/02/10/sharing-data-during-zika-and-other-global-health-emergencies/. Accessed 7 January 2017.

[3] Kaiser J. The preprint dilemma. Science. 29 September 2017. http://science.sciencemag.org/content/357/6358/1344. Accessed 30 September 2017.

[4] Lauer MS, Krumholz HM, Topol EJ. Time for a prepublication culture in clinical research? Lancet. 2015;386: 2447–2449. doi: 10.1016/S0140-6736(15)01177-0 26738703 10.1016/S0140-6736(15)01177-0 PMC5082701

[5] Vale RD. Accelerating scientific publication in biology. Proc Natl Acad Sci USA. 2015;112: 13439–13446. doi: 10.1073/pnas.1511912112 26508643 10.1073/pnas.1511912112 PMC4640799

[6] Bourne PE, Polka JK, Vale RD, Kiley R. Ten simple rules to consider regarding preprint submission. PLoS Comput Biol. 2017;13(5): e1005473 doi: 10.1371/journal.pcbi.1005473 28472041 10.1371/journal.pcbi.1005473 PMC5417409

[7] Smith R. Peer review: a flawed process at the heart of science and journals. J R Soc Med. 2006;99(4): 178–182. doi: 10.1258/jrsm.99.4.178 16574968 10.1258/jrsm.99.4.178 PMC1420798

[8] Open Science Collaboration. Estimating the reproducibility of psychological science. Science. 2015;349(6251): aac4716. doi: 10.1126/science.aac4716 26315443 10.1126/science.aac4716

[9] Lipsitch M, Finelli L, Heffernan RT, Leung GM, Redd SC. Improving the Evidence Base for Decision Making During a Pandemic: The Example of 2009 Influenza A/H1N1 Biosecur Bioterror. 2011;9: 89–115. 21612363 10.1089/bsp.2011.0007 PMC3102310

[10] Cauchemez S, Fraser C, Van Kerkhove MD, Donnelly CA, Riley S, Rambaut A, et al Middle East respiratory syndrome coronavirus: quantification of the extent of the epidemic, surveillance biases, and transmissibility. Lancet Infect Dis. 2014;14(1): 50–56. doi: 10.1016/S1473-3099(13)70304-9 24239323 10.1016/S1473-3099(13)70304-9 PMC3895322

